# Subclinical Risk Factors for Heart Failure With Preserved and Reduced Ejection Fraction Among Black Adults

**DOI:** 10.1001/jamanetworkopen.2022.31878

**Published:** 2022-09-15

**Authors:** Li Zhao, Rani Zierath, Jenine E. John, Brian Lee Claggett, Michael E. Hall, Donald Clark, Kenneth R. Butler, Adolfo Correa, Amil M. Shah

**Affiliations:** 1 Division of Cardiovascular Medicine, Brigham and Women’s Hospital, Boston, Massachusetts; 2 Division of Cardiovascular Medicine, the Sixth Medical Center, Chinese People’s Liberation Army General Hospital, Beijing, China; 3 University of Mississippi Medical Center, Jackson

## Abstract

**Importance:**

Sparse data exist regarding the contributions of subclinical impairments in cardiovascular and noncardiovascular function to incident heart failure (HF) with reduced ejection fraction (HFrEF) and preserved ejection fraction (HFpEF) among Black US residents, limiting understanding of the etiology of HF subtypes.

**Objectives:**

To identify subclinical cardiovascular and noncardiovascular risk factors associated with HFrEF and HFpEF in Black US residents.

**Design, Setting, and Participants:**

This cohort study used cross-sectional and time-to-event analysis with data from the community-based Jackson Heart Study (JHS), a longitudinal cohort study with baseline data collected from 2000 to 2004 (visit 1) and 10-year follow-up for incident HF. Black US residents from the Jackson, Mississippi, metropolitan area enrolled in JHS; those with prevalent HF, with moderate or greater aortic or mitral valve diseases on visit 1, who died before 2005, and who had missing HF status on follow-up were excluded. The analysis included 4361 participants and was performed between June 2020 to August 2021.

**Exposures:**

Quantitative measures of cardiovascular (left ventricular mass index [LVMI], left ventricular ejection fraction [LVEF], left atrial [LA] diameter, and pulse pressure) and noncardiovascular (percent predicted forced expiration volume in 1 second [FEV_1_ (percent predicted)], estimated glomerular filtration rate (eGFR), waist circumference, and hemoglobin A_1c_ [HbA_1c_] level) organ function.

**Main Outcomes and Measures:**

Incident HF, HFrEF, and HFpEF over 10-year follow-up.

**Results:**

The 4361 participants had a mean (SD) age of 54 (13); 2776 (64%) were women; and there were 163 HFpEF and 146 HFrEF events. In multivariable models incorporating measures reflecting each organ system, factors associated with incident HFpEF included greater LA diameter (hazard ratio [HR], 1.23; 95% CI, 1.03-1.47; *P* = .02), higher pulse pressure (HR, 1.23; 95% CI, 1.05-1.44; *P* = .009), lower FEV_1_ (percent predicted) (HR, 1.22; 95% CI, 1.04-1.43; *P* = .02), lower eGFR (HR, 1.43; 95% CI, 1.19-1.72; *P* < .001), higher HbA_1c_ level (HR, 1.25; 95% CI, 1.07-1.45; *P* = .005), and higher waist circumference (HR, 1.41; 95% CI, 1.18-1.69; *P* < .001). Factors associated with incident HFrEF included greater LVMI (HR, 1.25; 1.07-1.46; *P* = .005), lower LVEF (HR, 1.65; 95% CI, 1.42-1.91; *P* < .001), lower FEV_1_ (percent predicted) (HR, 1.19; 95% CI, 1.00-1.42; *P* = .047), and lower eGFR (HR, 1.27; 95% CI, 1.04-1.55; *P* = .02).

**Conclusions and Relevance:**

In this community-based cohort study of Black US residents, subclinical impairments in cardiovascular and noncardiovascular organ function were differentially associated with risk of incident HFpEF and HFrEF.

## Key Points

**Question:**

Are subclinical measures of cardiovascular and noncardiovascular organ function differentially associated with the incidence of heart failure with preserved ejection fraction (HFpEF) and heart failure with reduced ejection fraction (HFrEF)?

**Findings:**

In this cohort study of 4361 Black US residents, factors associated with incident HFpEF included left atrial diameter, pulse pressure, percent predicted forced expiration volume in 1 second, estimated glomerular filtration rate, and hemoglobin A_1c_ levels, while factors associated with incident HFrEF included left ventricular mass index, left ventricular ejection fraction, percent predicted forced expiration volume in 1 second, and estimated glomerular filtration rate. Measures of cardiovascular function were more strongly associated with incident HFrEF, while measures of noncardiovascular function were more strongly associated with incident HFpEF.

**Meaning:**

These findings suggest that partially distinct mechanisms may underlie HFrEF and HFpEF, with a greater contribution of multisystem noncardiac impairments to HFpEF.

## Teaser

This cohort study evaluates subclinical cardiovascular and noncardiovascular risk factors associated with heart failure with reduced and preserved ejection fraction among Black US residents in the Jackson Heart Study.

## Introduction

Compared with White US residents, Black US residents have a higher incidence of heart failure (HF), develop HF at a younger age, and experience worse outcomes once HF develops.^1,2,3,4,5,6^ Hypertension, coronary heart disease (CHD), diabetes, obesity, chronic kidney insufficiency, smoking, and unfavorable socioeconomic status are all established risk factors for HF.^7,8^ Both cardiovascular and noncardiovascular organ dysfunction contribute to the development of HF,^9^ and existing data suggest a greater contribution of noncardiovascular dysfunction to HF with preserved ejection fraction (HFpEF) compared with HF with reduced ejection fraction (HFrEF).^10^ Patterns of comorbidities and of alterations of cardiovascular structure and function are known to vary by race, as do metrics of social disadvantage.^3,5,11^ However, limited data exist regarding the relative contributions of impairments in cardiovascular and noncardiovascular organ function to the development of HFpEF and HFrEF in Black US residents and the extent to which these impairments account for the associations of social determinants of health with HF risk in this population.

We leveraged the detailed quantitative cardiovascular and noncardiovascular phenotyping and metrics of social adversity in Black participants enrolled in the Jackson Heart Study (JHS) to address these key knowledge gaps.^12^ We hypothesized that the relative contributions of cardiovascular and noncardiovascular dysfunction to incident HFpEF and HFrEF would differ, and that evaluating their prospective associations with incident HF could provide insights into the etiologic contributors to HF in this population.

## Methods

### Study Population

The Jackson Heart Study is an ongoing epidemiology study of Black US residents that recruited 5306 participants from 3 counties (Hinds, Madison, and Rankin) of the Jackson, Mississippi, metropolitan area in 2000 to 2004 (visit 1). Race was categorized based on self-report from Mississippi driver’s licenses or census data.^13^ Detailed measurements of anthropometry, vital signs, prevalent cardiovascular and noncardiovascular diseases, health behaviors, medication use, laboratory tests, and echocardiographic measurements were assessed at baseline.^12^ The JHS protocol was approved by the institutional review boards of Jackson State University, Tougaloo College, and the University of Mississippi Medical Center in Jackson, Mississippi. All study participants provided written informed consent. This study followed the Strengthening the Reporting of Observational Studies in Epidemiology (STROBE) reporting guideline.

Participants with prevalent HF at visit 1 (n = 141) or with moderate or greater aortic or mitral valve diseases on visit 1 echocardiogram (n = 143) were excluded from this analysis. Prevalent HF at visit 1 was defined based on self-report of HF on the first annual telephone follow-up call after visit 1 (mean [SD] time from visit 1 to follow-up call, 1.1 [0.3] years). An additional 216 participants who died before initiation of HF surveillance and adjudication in 2005 were also excluded. After excluding 445 participants with missing HF status on follow-up, 4361 participants were included for the current analysis. All measures were assessed at visit 1.

### Clinical Covariates

CHD was defined based on self-reported history of myocardial infarction and/or electrocardiogram (EKG) evidence of a prior myocardial infarction (per Minnesota code), as previously described.^14^ Hypertension status was defined as diastolic blood pressure greater than 140 mm Hg and systolic blood pressure greater than 90 mm Hg (per Seventh Report of the Joint National Committee on Prevention, Detection, and Treatment of High Blood Pressure) or use of blood pressure–lowering medication at visit 1.^15^ Pulse pressure was defined as the difference between systolic and diastolic blood pressures.^12^ Diabetes was defined as a hemoglobin A_1c_ (HbA_1c_) level of 6.5% or greater (to convert to proportion of total hemoglobin multiply, by 0.01), a fasting plasma glucose level of 126 mg/dL or greater (to convert to micromoles per liter, multiply by 0.0555), or a self-report of diabetes medication use, as previously described.^16^ Cigarette smoking was derived from participant interview.^17^

### Echocardiographic Measures of Cardiac Structure and Function

Echocardiograms were recorded by trained sonographers at visit 1 and interpreted by experienced cardiologists in the Echocardiography Reading Center at the University of Mississippi Medical Center (eMethods in the Supplement).^18^ The JHS Echocardiography Manual is available on the JHS website, as previously described.^19^

### Measures of Noncardiovascular Organ Function

Body mass index was calculated as weight in kilograms divided by height in meters squared, and waist circumference was measured in centimeters. Percent predicted values for maximum forced expiratory volume at 1 second (FEV_1_ [percent predicted]) and forced vital capacity (FVC [percent predicted]) were calculated using appropriate reference equations.^20,21^ Estimated glomerular filtration rate (eGFR) was calculated based on serum creatinine using the Chronic Kidney Disease Epidemiology equation.^22^ Details regarding measurement of plasma adiponectin, leptin, HbA_1c_, and high-sensitivity C-reactive protein (hsCRP) are provided in the eMethods in the Supplement.

### Measures of Social Determinants of Health

Economic status was categorized into 4 levels based on household income, family size, and US census poverty.^23^ Educational attainment was based on self-report of years of schooling completed.^23^ The neighborhood problems scale comprised 6 items scored from 1 (not really a problem) to 4 (very serious problem), as previously described.^24^ The eMethods in the Supplement includes further details.

### Incident HF and Death Events

Surveillance for incident HF hospitalization started from January 1, 2005. Ascertainment consisted of surveillance of hospital discharge and death certificate keywords and *International Classification of Diseases, Ninth Revision *(*ICD-9*) and *International Statistical Classification of Diseases and Related Health Problems, Tenth Revision *(*ICD-10*) codes suggestive of HF, as previously described.^25^ For potential HF events, hospitalization medical records were abstracted and underwent physician adjudication. Incident HFpEF or HFrEF was based on the presence of an adjudicated HF event with a documented left ventricular ejection fraction (LVEF) of 50% or greater for HFpEF or less than 50% for HFrEF from medical record abstraction during the incident HF hospitalization, similar to the approach used in the Atherosclerosis Risk in Communities study.^26^

### Statistical Analysis

The analysis was performed between June 2020 to August 2021. Baseline characteristics were compared between those who developed incident HF vs those who did not during follow-up, with *t* test or χ^2^ test.

The association of each measure of cardiovascular and noncardiovascular organ function with incident HF overall, HFpEF, and HFrEF was assessed using multivariable Cox proportional hazard regression models adjusting for age, sex, hypertension, diabetes, smoking status, and CHD (myocardial infarction) history. The following systems were considered: cardiovascular, left ventricular (LV) structure, LV systolic function, LV diastolic function, and systemic arterial function; noncardiovascular, pulmonary function, kidney function, body composition, dysglycemia, and inflammation (eTable 1 in the Supplement). To facilitate comparability between factors associated with the outcome, LVEF was modeled as negative LVEF, such that higher values would be associated with higher risk. To assess the extent to which an organ system was associated with HF while accounting for the other systems, one measure was selected to represent each system based on the significance of association with incident HF and less than 5% missing values. Hazard ratios (HRs) of cardiovascular and noncardiovascular functional measures in models for risk of HFpEF vs those for risk of HFrEF were compared with Poisson regression models. Sensitivity analyses were performed using multiple imputation by chained equations, an iterative imputation procedure (mi impute chained in Stata), to account for missing data (eMethods in the Supplement).^27^

Using variables that remained significantly associated with HF in models, including representative measures from all considered organ systems, we used standardized coefficients from the multivariable models to generate a cardiovascular (CV) score that included LV mass index (LVMI), LVEF, left arterial diameter (LAD), and pulse pressure, and a noncardiovascular (non-CV) score that included FEV_1_ (percent predicted), eGFR, HbA_1c_ level, and waist circumference. Potential effect modification by sex was assessed using multiplicative interaction terms. The association between measures of social determinants of health and incident HF were evaluated in multivariable Cox regression models adjusting for demographic characteristics and comorbidities. Further adjustment was then performed for the CV score, non-CV score, or both.

Stata version 16.0 (StataCorp) was used for the analysis, and R i386 version 4.1.0 (R Project for Statistical Computing) was used to generate components of the figures. For all the analyses, a 2-sided *P* < .05 was considered statistically significant.

## Results

### Baseline Characteristics

The mean (SD) age of the 4361 participants was 54 (13) years old, 2776 (64%) were women, hypertension was prevalent in 2368 (54%), and 958 (22%) had diabetes. Over a median follow-up of 10 years, 348 developed incident HF (163 HFpEF, 146 HFrEF, and 39 with unknown LVEF). Compared with participants who did not develop HF, those who developed HF were older and had a higher prevalence of cardiovascular risk factors, including hypertension, diabetes, prior myocardial infarction, and smoking (Table 1). They also had lower economic status, lower education attainment, and higher measures of neighborhood problems. Those who developed HF had greater LV wall thickness and LVMI, lower LVEF, greater LA size, higher pulse pressure, greater waist circumference, lower eGFR, and worse pulmonary function reflected in FEV_1_ (percent predicted), FVC (percent predicted), and FEV_1_/FVC ratio.

**Table 1.** Cardiovascular and Noncardiovascular Characteristics by Incident HF Subtypes

Characteristic	Missing, %	Mean (SD)			
		No HF (n = 4013)	Any HF (n = 348)	HFpEF (n = 163)	HFrEF (n = 146)
Age, y	0	54 (12)	64 (11)^a^	65 (11)^a^	62 (12)^a^^,^^b^
Gender, No. (%)					
Male	0	1458 (36.3)	127 (36.5)	45 (27.6)^a^	71 (48.6)^a^^,^^b^
Female	0	2465 (63.7)	221 (63.5)	118 (72.4)^a^	75 (51.4)^a^^,^^b^
LV structure					
LVEDD, mm	5.0	48.1 (4.1)	49.1 (5.1)^a^	47.9 (4.5)	50.6 (5.3)^a^^,^^b^
MWT, mm	5.0	8.7 (1.3)	9.3 (1.5)^a^	9.4 (1.6)^a^	9.2 (1.6)^a^
RWT	5.0	0.36 (0.06)	0.37 (0.08)^a^	0.39 (0.09)^a^	0.36 (0.07)^b^
LVMI, g/m^2^	5.1	72.2 (17.4)	81.1 (21.3)^a^	78.8 (19.6)^a^	83.2 (22.7)^a^
LV systolic function					
LVEF, %	4.1	63.4 (7.7)	61.5 (9.3)^a^	64.3 (7.1)^a^	57.9 (10.2)^a^^,^^b^
LV diastolic function					
LA diameter, mm	5.1	34.8 (4.2)	36.5 (5.1)^a^	36.7 (4.6)^a^	36.3 (5.9)^a^
E/A ratio	10.3	1.11 (0.34)	0.95 (0.29)^a^	0.94 (0.29)^a^	0.97 (0.31)^a^
Systemic arterial function					
Hypertension, No. (%)	0	2093 (52.2)	275 (79.0)^a^	136 (83.4)^a^	108 (74.0)^a^^,^^b^
Using hypertension medication, No. (%)	0.8	1910 (48.0)	261 (75.4)^a^	131 (80.4)^a^	100 (69.0)^a^^,^^b^
SBP, mm Hg	0.4	126 (16)	134 (19)^a^	135 (20)^a^	133 (17)^a^
Pulse pressure, mm Hg	0.4	50 (14)	60 (17)^a^	62 (17)^a^	58 (16)^a^^,^^b^
Pulmonary function					
FEV_1_ (percent predicted), %	5.5	93 (17)	87 (19)^a^	86 (20)^a^	88 (19)^a^
FVC (percent predicted), %	5.5	92 (17)	88 (21)^a^	86 (19)^a^	89 (23)
FEV_1_/FVC ratio	5.5	0.81 (0.08)	0.78 (0.09)^a^	0.79 (0.09)^a^	0.77 (0.10)^a^
Kidney function					
eGFR, mL/min/1.73 m^2^	1.6	97 (20)	83 (26)^a^	81 (25)^a^	86 (27)^a^
Dysglycemia					
Diabetes, No. (%)	1.1	787 (19.8)	171 (49.6)^a^	86 (53.4)^a^	68 (46.9)^a^
HbA_1c _level, %	3.7	5.9 (1.2)	6.7 (1.9)^a^	6.8 (2.0)^a^	6.6 (2.0)^a^
Using diabetes medication, No. (%)	0.9	492 (12.4)	135 (39.1)^a^	69 (42.6)^a^	52 (35.9)^a^
Obesity related					
BMI	0.2	31.6 (7.0)	32.8 (7.0)^a^	34.2 (7.6)^a^	31.3 (6.2)^b^
Waist circumference, cm	0.2	100 (16)	106 (16)^a^	109 (16)^a^	104 (16)^a^^,^^b^
Adiponectin level, median (IQR), g/mL	3.2	4119 (2656-6509)	4827 (3066-8567)^a^	4987 (3043-8934)^a^	4489 (3050-6841)
Leptin level, median (IQR), ng/mL	2.5	22.7 (10.2-38.8)	25.0 (10.4-43.6)^a^	30.9 (16.2-51.8)^a^	19.5 (7.9-33.4)^a^^,^^b^
Inflammation					
hsCRP level, median (IQR), mg/dL	1.8	0.026 (0.010-0.055)	0.033 (0.014-0.066)^a^	0.039 (0.018-0.071)^a^	0.028 (0.012-0.065)^b^
Smoking status, No. (%)					
Ever	0.2	1211 (30.2)	142 (40.9)^a^	68 (41.7)^a^	61 (42.1)^a^
Disease history, No. (%)					
CHD or MI history	0	169 (4.2)	44 (12.6)^a^	19 (11.7)^a^	20 (13.7)^a^
Social determinations of health					
Income status, No. (%)					
Affluent	15.4	451 (13.2)	59 (20.3)^a^	30 (22.2)^a^	22 (17.9)^a^
Upper-middle	15.4	783 (23.0)	103 (35.5)^a^	51 (37.8)^a^	44 (35.8)^a^
Lower-middle	15.4	1040 (30.5)	72 (24.8)^a^	30 (22.2)^a^	33 (26.8)^a^
Poor	15.4	1133 (33.3)	56 (19.3)^a^	24 (17.8)^a^	24 (19.5)^a^
Education attainment categorization, No. (%)					
Attended vocational school, trade school, or college	0.3	603 (15.1)	137 (39.5)^a^	66 (40.5)^a^	51 (35.2)^a^
High school graduate or GED	0.3	789 (19.7)	70 (20.2)^a^	33 (20.2)^a^	30 (20.7)^a^
Less than high school	0.3	2609 (65.2)	140 (40.3)^a^	64 (39.3)^a^	64 (44.1)^a^
Neighborhood problems	0.3	1.56 (0.19)	1.62 (0.17)^a^	1.65 (0.17)^a^	1.59 (0.17)^a^^,^^b^

Abbreviations: BMI, body mass index (calculated as weight in kilograms divided by height in meters squared); CHD, coronary heart disease; eGFR, estimated glomerular filtration rate; FEV_1_, forced expiration volume in 1 second; FVC, forced vital capacity; HbA_1c_, hemoglobin A_1c_; HF, heart failure; HFpEF, HF with preserved ejection fraction; HFrEF, HF with reduced ejection fraction; hsCRP, high-sensitivity C-reactive protein; LA, left atrial; LV, left ventricular; LVEDD, left ventricular end-diastolic dimension; LVEF, left ventricular ejection fraction; LVMI, left ventricular mass index; MI, myocardial infarction; MWT, mean wall thickness; RWT, relative wall thickness; SBP, systolic blood pressure.

SI conversion factors: To convert HbA_1c _to proportion of total hemoglobin, multiply by 0.01; hsCRP to milligrams per liter, multiply by 10.

^a^ *P* < .05 vs no HF group.

^b^ *P* < .05 vs HFpEF group.

Among participants who developed HF, those who developed HFpEF were older, more likely to be female, and more frequently had hypertension than those who developed HFrEF. They also had smaller LV dimensions despite higher BMI and waist circumference, higher LVEF, and greater pulse pressure (Table 1). These trends were similar within each sex category (eTables 2 and 3 in the Supplement).

### Factors Associated With Incident HF

In multivariable Cox regression models adjusted for age, sex, hypertension, diabetes, smoking status, and CHD history, multiple echocardiographic and noncardiac organ function measures were associated with incident HF (Table 2). Similar results were observed in analyses additionally adjusting for income level and education attainment (eTable 4 in the Supplement). Based on magnitude and strength of association with incident HF in these models, the following measures were included in a single multivariable model incorporating measures reflecting each organ system: LVMI (representing LV structure), LVEF (LV systolic function), LAD (diastolic function), FEV_1_ (percent predicted) (pulmonary function), eGFR (kidney function), pulse pressure (systemic arterial function), HbA_1c_ level (dysglycemia), waist circumference (body composition), and hsCRP level (inflammation). In this model, all measures remained significantly associated with the risk of HF except hsCRP, which was subsequently removed from the final model (eTable 5 in the Supplement). The standardized HRs were similar in magnitude for each measure, with the largest magnitude noted for LVEF and eGFR. Similar results were observed in sensitivity analyses using multiple imputation for missing data (eTable 6 in the Supplement).

**Table 2.** Associations of Cardiovascular and Noncardiovascular Measures With Incident HF Events in Unifactor Multivariable Models

Factor	Mean (SD)		HR for incident HF^a^	
	No HF (n = 4013)	HF (n = 348)	HR (95% CI)	*P* value
LV structure				
LVEDD, mm	48.1 (4.1)	49.1 (5.1)^b^	1.28 (1.15-1.41)	<.001
MWT, mm	8.7 (1.3)	9.3 (1.5)^b^	1.17 (1.05-1.29)	.02
RWT	0.36 (0.06)	0.37 (0.08)^b^	1.02 (0.92-1.13)	.76
LVMI, g/m^2^	72.2 (17.4)	81.1 (21.3)^b^	1.21 (1.11-1.32)	<.001
LV systolic function				
LVEF, %	63.4 (7.7)	61.5 (9.3)^b^	1.32 (1.20-1.45)	<.001
LV diastolic function				
LA diameter, mm	34.8 (4.2)	36.5 (5.1)^b^	1.29 (1.17-1.43)	<.001
E/A ratio	1.11 (0.34)	0.95 (0.29)^b^	0.99 (0.85-1.15)	.90
Systemic arterial function				
SBP, mm Hg	126 (16)	134 (19)^b^	1.19 (1.07-1.32)	.001
Pulse pressure, mm Hg	50 (14)	60 (17)^b^	1.24 (1.12-1.37)	<.001
Pulmonary function				
FEV_1_ (percent predicted), %	93 (17)	87 (19)^b^	1.26 (1.13-1.40)	<.001
FVC (percent predicted), %	92 (17)	88 (21)^b^	1.17 (1.04-1.31)	.006
FEV_1_/FVC ratio	0.81 (0.08)	0.78 (0.09)^b^	1.13 (1.02-1.24)	.02
Kidney function				
eGFR, mL/min/1.73 m^2^	97 (20)	83 (26)^b^	1.28 (1.13-1.45)	<.001
Dysglycemia related				
HbA_1c_ level, %	5.9 (1.2)	6.7 (1.9)^b^	1.22 (1.11-1.35)	<.001
Obesity related				
BMI	31.6 (7.0)	32.8 (7.0)^b^	1.23 (1.10-1.38)	<.001
Waist circumference, cm	100 (16)	106 (16)^b^	1.33 (1.19-1.48)	<.001
Adiponectin, ng/mL	4119 (2656-6509)	4827 (3066-8567)^b^	1.20 (1.08-1.35)	.001
Leptin, ng/mL	22.7 (10.2-38.8)	25.0 (10.4-43.6)^b^	1.17 (1.00-1.38)	.05
Inflammation				
hsCRP, mg/L	0.26 (0.10-0.55)	0.33 (0.14-0.66)^b^	1.17 (1.05-1.32)	.006

Abbreviations: BMI, body mass index (calculated as weight in kilograms divided by height in meters squared); eGFR, estimated global filtration rate; FEV_1_, forced expiration volume in 1 second; FVC, forced vital capacity; HbA_1c_, hemoglobin A_1c_; HF, heart failure; HR, hazard ratio; hsCRP, high sensitivity C-reactive protein; LA, left atrial; LV, left ventricular; LVEDD, left ventricular end-diastolic dimension; LVEF, left ventricular ejection fraction; LVMI, left ventricular mass index; MWT, mean wall thickness; RWT, relative wall thickness; SBP, systolic blood pressure.

SI conversion factors: To convert HbA_1c _to proportion of total hemoglobin, multiply by 0.01; hsCRP to milligrams per liter, multiply by 10.

^a^ The models were adjusted for age, sex, hypertension, diabetes, smoking status, and coronary heart disease (myocardial infarction) history. All continuous variables were standardized. The HRs for LVEF, FEV_1_ (percent predicted), FVC (percent predicted), FEV1/FVC ratio, and eGFR were inverted to facilitate the comparison of the magnitude of associations of the cardiovascular and noncardiovascular measures with the outcome. HRs for adiponectin, leptin, and hsCRP were per 1-unit increase of log-transformed forms.

^b^ *P* < .05 vs no HF group.

### Associations of Cardiovascular and Noncardiovascular Dysfunction With Incident HFpEF and HFrEF

**Figure 1.**
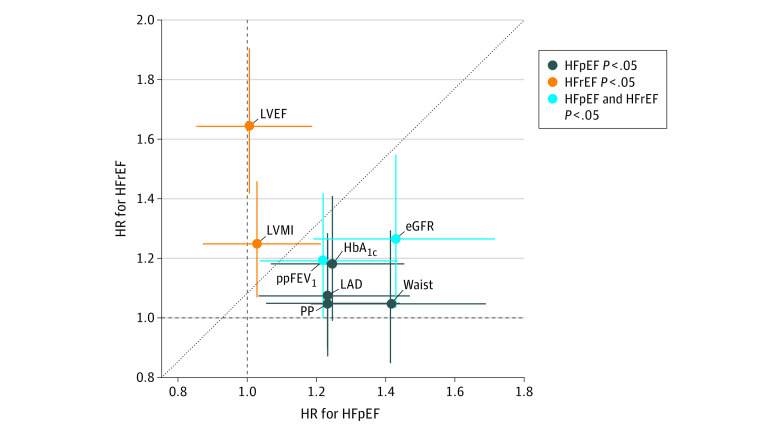
Cardiovascular and Noncardiovascular Factors Associated With Incident Heart Failure With Preserved Ejection Fraction (HFpEF) and Heart Failure With Reduced Ejection Fraction (HFrEF) The standardized hazard ratio (HR)–HR plot shows independent factors associated with HFrEF, including left ventricular ejection fraction (LVEF) and left ventricular mass index (LVMI), and for HFpEF, including left atrial diameter (LAD), pulse pressure (PP), hemoglobin A_1c_ (HbA_1c_) level, and waist circumference (waist), and for both HFrEF and HFpEF, including estimated glomerular filtration rate (eGFR) and percent predicted forced expiration volume in 1 second (ppFEV_1_).

In models including measures representing each considered organ system, significant factors associated with incident HFpEF included greater LAD (HR, 1.23; 95% CI, 1.03-1.47; *P* = .02), higher pulse pressure (HR, 1.23; 95% CI, 1.05-1.44; *P* = .009), lower FEV_1_ (percent predicted) (HR, 1.22; 95% CI, 1.04-1.43; *P* = .02), lower eGFR (HR, 1.43; 95% CI, 1.19-1.72; *P* < .001), higher HbA_1c_ level (HR, 1.25; 95% CI, 1.07-1.45; *P* = .005), and greater waist circumference (HR, 1.41; 95% CI, 1.18-1.69; *P* < .001) (Figure 1; eTable 5 and eFigure 2 in the Supplement). Factors associated with incident HFrEF included greater LVMI (HR, 1.25; 1.07-1.46; *P* = .005), lower LVEF (HR, 1.65; 95% CI, 1.42-1.91; *P* < .001), lower FEV_1_ (percent predicted) (HR, 1.19; 95% CI, 1.00-1.42; *P* = .047), and lower eGFR (HR, 1.27; 95% CI, 1.04-1.55; *P* = .02). Lower FEV_1_ (percent predicted) and eGFR were independent factors associated with both HFpEF and HFrEF. In sensitivity analysis excluding 240 participants with a baseline LVEF of less than 50%, LVEF remained a significant factor associated with incident HFrEF but not incident HFpEF (eTable 7 in the Supplement).

Assessment for possible effect modification of sex on the association of organ function measures with incident HFpEF or HFrEF suggested a possible larger association of lower eGFR with incident HFrEF in women compared with men (women: HR per SD, 1.53; 95% CI, 1.20-1.96; men: HR per SD, 0.95; 95% CI, 0.68-1.32; *P *for interaction = .03) (eTables 8 and 9 in the Supplement). No other effect modification by sex was observed. Given the large number of comparisons and marginal interaction *P* values, this potential interaction should be interpreted with caution.

### Socioeconomic Measures and Incident HF

**Figure 2.**
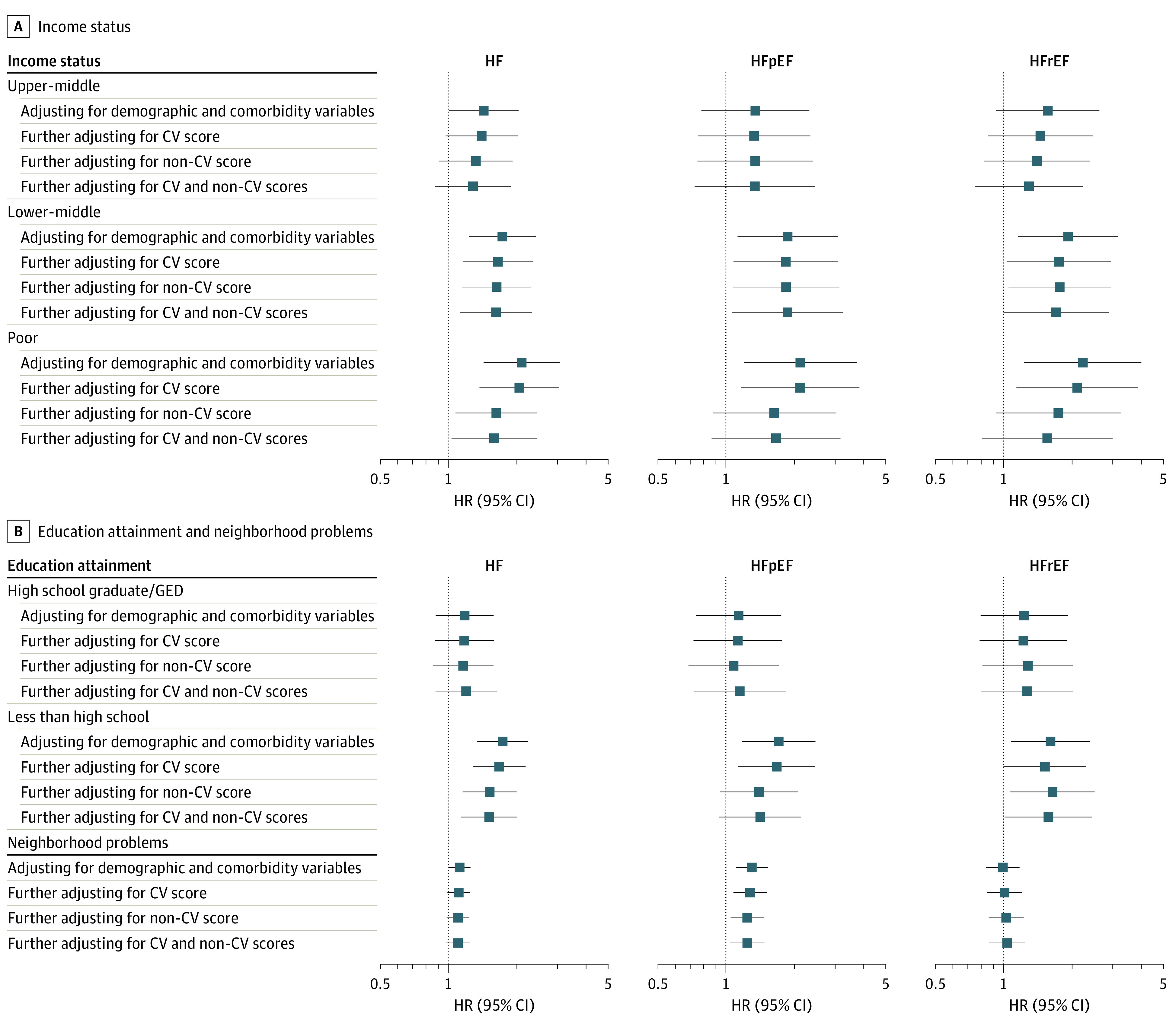
Associations of Socioeconomic Metrics With Incident Heart Failure (HF) Events The associations of some socioeconomic measures (income status and education attainment) with incident HF or HF subtypes (adjusted for age, sex, hypertension, diabetes, smoking status, and coronary heart disease history) were attenuated when further adjusted for cardiovascular (CV) risk score, non-CV score, or both. The reference level for income status was affluent, and the reference level for education attainment was vocational school, trade school, or college. HR indicates hazard ratio; HFpEF, heart failure with preserved ejection fraction; HFrEF, heart failure with reduced ejection fraction.

In analyses adjusting for demographics and comorbidities, low-middle and poor income status were associated with incident HF, with similar magnitudes of association with incident HFpEF and HFrEF (Table 3). Less than high school education was associated with incident HF, while neighborhood problems were only associated with incident HFpEF. In models that further adjusted for CV and non-CV risk scores, the association of low-middle and poor income status with incident HF was attenuated. In contrast, the associations of low education attainment and neighborhood problems with incident HF and HFpEF, respectively, were only modestly attenuated and remained significant (low education: HR, 1.50; 95% CI, 1.13-1.99; *P* = .005; neighborhood problems: 1.23; 95% CI, 1.04-1.47; *P* = .02) (Figure 2).

**Table 3.** Association of Socioeconomic Variables With Incident HF^a^

Factor	HF overall		HFpEF		HFrEF		*P* value for HFrEF vs HFpEF
	HR (95% CI)	*P* value	HR (95% CI)	*P* value	HR (95% CI)	*P* value	
Income status							
Affluent	1 [Reference]	NA	1 [Reference]	NA	1 [Reference]	NA	NA
Upper-middle	1.42 (1.00-2.02)	.05	1.34 (0.78-2.31)	.30	1.58 (0.93-2.68)	.09	.67
Lower-middle	1.72 (1.22-2.40)	.002	1.85 (1.12-3.07)	.02	1.94 (1.16-3.24)	.01	.90
Poor	2.09 (1.42-3.06)	<.001	2.11 (1.19-3.73)	.01	2.26 (1.24-4.11)	.008	.87
Education attainment categorization							
Attended vocational school, trade school, or college	1 [Reference]	NA	1 [Reference]	NA	1 [Reference]	NA	NA
High school graduate/GED	1.17 (0.87-1.57)	.29	1.13 (0.73-1.74)	.58	1.22 (0.79-1.90)	.37	.82
Less than high school	1.72 (1.33-2.22)	<.001	1.69 (1.17-2.45)	.005	1.60 (1.07-2.39)	.02	.86
Neighborhood problems (age & sex adjusted), per SD	1.11 (1.00-1.24)	.05	1.29 (1.10-1.52)	.002	0.99 (0.83-1.17)	.89	.02

Abbreviations: HF, heart failure; HFpEF, HF with preserved ejection fraction; HFrEF, HF with reduced ejection fraction; HR, hazard ratio.

^a^ The models were adjusted for age, sex, hypertension, diabetes, smoking status, and coronary heart disease (myocardial infarction) history.

## Discussion

In this large community-based cohort of Black US residents free of prevalent HF and followed up for a median of 10 years, subclinical impairments in both cardiovascular and noncardiovascular organ function were differentially associated with risk of incident HFpEF and HFrEF. While cardiac structural (LVMI) and systolic (LVEF) measures were associated with incident HFrEF, measures reflecting LV diastolic function (LAD) and vascular stiffness (pulse pressure) were associated with incident HFpEF, as were several measures of noncardiovascular organ function (HbA_1c_ level, waist circumference). Lower eGFR and FEV_1_ (percent predicted) were associated with both incident HFpEF and HFrEF. Measures of social adversity were significantly associated with HF risk. While cardiovascular and noncardiovascular organ function partially accounted for the association of worse economic status with HF risk, they did not attenuate associations of lower educational attainment and greater neighborhood problems with incident HF and HFpEF, respectively. These findings support partially distinct mechanisms underlying HFrEF and HFpEF, and additional mechanisms not captured by the subclinical measures considered by which social adversity affects HF risk.

HF is a multisystem disorder characterized by both cardiac dysfunction and impairment in several noncardiac organs. Data regarding the prospective association of measures of cardiovascular and noncardiovascular organ function with incident HFpEF or HFrEF are limited, particularly in Black adults. Our finding of a differential association of higher LVMI with risk of incident HFrEF is consistent with prior findings from the multicohort International Collaboration on Heart Failure Subtypes, which similarly observed a differential association of EKG-based LVH with incident HFrEF as opposed to HFpEF.^28^ Similarly, our finding of a differential association of lower LVEF with incident HFrEF but not HFpEF extends previous findings from the predominantly White Framingham Heart Study of a differential association of LV systolic dysfunction, defined by visual estimation on transthoracic echocardiograms, with incident HFrEF.^10^ The differential association of greater LA size with risk of incident HFpEF similarly extends to a more diverse population previous findings of a differential association of LV diastolic dysfunction—defined primarily by transmitral Doppler measures—with incident HFpEF in that same study.

Beyond LV systolic and diastolic function, impairments in systemic arterial function and functional reserve have been implicated in the pathophysiology of exercise intolerance in HFpEF.^29,30^ However, despite the high prevalence of hypertension in HFpEF, previous studies have not observed a differential association of systolic BP or antihypertensive therapy with risk of HFpEF compared with HFrEF.^28^ Our findings that higher pulse pressure—a metric of arterial stiffness^31^—is differentially associated with risk of HFpEF supports a potential role for systemic arterial dysfunction in the development of HFpEF.

Cross-sectional data from HF registries suggest a higher burden of noncardiac comorbidities among patients with HFpEF compared with HFrEF, including COPD, obesity, and possibly diabetes.^32,33^ Notably, the prevalence of kidney dysfunction in registry studies appears similar among patients with HFpEF and HFrEF. Despite these consistent associations across several registries and clinical trial samples, limited data exist regarding the associations of subclinical impairments in noncardiac organ function with the development of HFpEF or HFrEF. The lack of prospective data are important, as the interpretation of cross-sectional associations is confounded by survivor bias and possible reverse causation given the multisystem impact of HF once it develops. Our findings from a large community-based sample of Black US residents support the contribution of distinct cardiovascular and diverse noncardiovascular impairments to the development of HFpEF compared with HFrEF. These results are consistent with the observation of a greater proportion of adverse outcomes, such as death, being noncardiovascular in origin among patients with HFpEF.^34^ Given that HFpEF is becoming the predominant HF subtype, a comprehensive measure targeting systematic organ dysfunctions might be necessary to reduce the risk of developing HFpEF as well as HF overall.

Prior studies of the association of subclinical impairments in kidney function with risk of HF phenotype have been conflicting,^35^ and we observed significant associations of lower eGFR with both incident HFpEF and HFrEF. Previous data from the Framingham Heart Study demonstrated an association of lower ratio of FEV_1_ (percent predicted) to FVC with risk of incident HFpEF but not HFrEF. In contrast, in our analysis, FEV_1_ (percent predicted) was the spirometric variable most robustly associated with HF risk and associated with risk of both HFpEF and HFrEF. The importance of obesity in the pathophysiology and management of HFpEF has recently been emphasized.^36,37,38^ Despite the higher prevalence of obesity in the cohort with HFpEF compared with that with HFrEF,^32,39^ higher BMI was associated with greater risk of both incident HFpEF and HFrEF without robust findings to suggest differential association in the International Collaboration on Heart Failure Subtypes.^28^ While higher BMI was associated with greater risk of HF in our study, greater waist circumference was more robustly associated with HF and was independently associated with incident HFpEF, while having no association with incident HFrEF. This was in accordance with previous findings that visceral adipose tissue instead of subcutaneous adipose tissue was associated with incident HFpEF, while no single marker of obesity was related to incident HFrEF.^40^

Most data regarding risk factors and functional impairments associated with incident HFpEF and HFrEF originate from studies of predominantly White communities that may not generalize to other communities with greater burdens of social adversity.^10,28^ Social determinants of health, including low socioeconomic status, are associated with greater comorbidity burden and risk of cardiovascular events.^41,42^ A unique strength of the current analysis is associating measures of cardiovascular and noncardiovascular dysfunction with HF risk in the context of measures of social adversity. After adjusting for demographics and cardiovascular comorbidities, lower-middle and poor income status and less than high school education attainment were associated with risk of both HFpEF and HFrEF, while neighborhood problems were more robustly associated with incident HFpEF compared with HFrEF. Furthermore, associations of low-income status with all HF end points were not significant after adjustment for cardiovascular and noncardiovascular scores, while low education attainment and neighborhood problems remained significantly associated with HF end points. These findings suggest potentially distinct mechanisms by which different social determinants of health may influence HF risk, and they warrant further investigation.

### Limitations

This analysis has several limitations. HF status was not collected in JHS at visit 1, and prevalent HF had to be defined based on self-report at the closest annual follow-up call. While previous data suggest good specificity of self-report when compared with physician-diagnosed HF,^43^ the sensitivity is limited and may have resulted in some participants with prevalent HF at visit 1 being included in our analysis. Furthermore, HF surveillance and adjudication started only after January 2005. Therefore, incident HF events after visit 1 but before January 2005 (mean [SD] time, 2.4 [0.8] years) were not captured. However, the delayed initiation of follow-up relative to visit 1 assessments may help minimize the risk of reverse causation due to prevalent but not clinically declared HF at the time of visit 1 quantitative assessments. Follow-up HF status was missing in 445 participants excluded from this analysis, which may potentially bias the results. Not all contemporary measures of cardiovascular and noncardiovascular organ system functions were available in the current analysis, most notably contemporaneous measures of LV diastolic (tissue Doppler e′, E/e′ ratio, LA volume index) and systolic (LV longitudinal and circumferential strains) function. Although this may limit the sensitivity of our cardiac function assessments, the LV function measures used have been prognostically validated and widely used to estimate HF risk. Health system–related factors, such as medical care and organization of medical services, can importantly affect the risk of developing HF, but data on these factors at JHS visit 1 were limited and therefore are not accounted for in this analysis. As in all observational studies, residual confounding is a limitation in that unmeasured variables could explain the outcome of HF. Also, there were multiple comparisons in our analysis that could increase the likelihood of false-positive results.

## Conclusions

In this study, subclinical impairments in both cardiovascular and noncardiovascular organ function were differentially associated with risk of incident HFpEF and HFrEF. While cardiac structural and systolic measures were associated with incident HFrEF, measures reflecting LV diastolic function and vascular stiffness were associated with incident HFpEF, as were several measures of noncardiovascular organ function (ie, HbA_1c_ level, waist circumference). These findings support partially distinct mechanisms underlying HFrEF and HFpEF, and the association of adverse socioeconomic status with incident HF might be partly explained by subclinical CV and non-CV organ dysfunctions.

## Notes

Supplement.
eMethods.
**eFigure 1. **Study Population Flow Chart **eFigure 2.** Associations of Cardiovascular and Noncardiovascular Dysfunctions with Incident HF **eTable 1.** Systems Evaluated in Risk Models for Heart Failure **eTable 2. **Cardiovascular and Noncardiovascular Characteristics by Incident HF Subtypes in Women **eTable 3. **Cardiovascular and Noncardiovascular Characteristics by Incident HF Subtypes in Men **eTable 4.** Associations of Cardiovascular and Noncardiovascular Measures With Incident HF Events in Unipredictor Multivariable Models Additionally Adjusting For Income Status and Education Attainment **eTable 5. **Associations of Cardiovascular and Noncardiovascular Measures With Incident HF Events in Multipredictor Models **eTable 6. **Cox Regression Models for HF, HFpEF, and HFrEF with Multiple Imputation **eTable 7.** Associations of Cardiovascular and Noncardiovascular Measures With Incident HF Events in Multipredictor Models Excluding Participants With a Baseline LVEF Less Than 50% **eTable 8.** Exploration of Potential Interactions of Organ Function Measures with Sex for Incident HF **eTable 9. **Stratified Cox Regression Models for HF, HFpEF, and HFrEF by Sex Categories
eReferences.
