# Supplementary material for: Subclinical Risk Factors for Heart Failure With Preserved and Reduced Ejection Fraction Among Black Adults
Source: JAMA Netw Open. 2022 Sep 15;5(9):e2231878. doi: 10.1001/jamanetworkopen.2022.31878 (PMC9478780; doi:10.1001/jamanetworkopen.2022.31878)
Supplement: Supplement. — eMethods. eFigure 1. Study Population Flow Chart eFigure 2. Associations of Cardiovascular and Noncardiovascular Dysfunctions with Incident HF eTable 1. Systems Evaluated in Risk Models for Heart Failure eTable 2. Cardiovascular and Noncardiovascular Characteristics by Incident HF Subtypes in Women eTable 3. Cardiovascular and Noncardiovascular Characteristics by Incident HF Subtypes in Men eTable 4. Associations of Cardiovascular and Noncardiovascular Measures With Incident HF Events in Unipredictor Multivariable Models Additionally Adjusting For Income Status and Education Attainment eTable 5. Associations of Cardiovascular and Noncardiovascular Measures With Incident HF Events in Multipredictor Models eTable 6. Cox Regression Models for HF, HFpEF, and HFrEF with Multiple Imputation eTable 7. Associations of Cardiovascular and Noncardiovascular Measures With Incident HF Events in Multipredictor Models Excluding Participants With a Baseline LVEF Less Than 50% eTable 8. Exploration of Potential Interactions of Organ Function Measures with Sex for Incident HF eTable 9. Stratified Cox Regression Models for HF, HFpEF, and HFrEF by Sex Categories eReferences. [file jamanetwopen-e2231878-s001.pdf]

Supplemental Online Content

Zhao L, Zierath R, John JE, et al. Subclinical risk factors for heart failure with preserved and reduced ejection fraction among Black adults. *JAMA Netw Open.* 2022;5(9):e2231878. doi:10.1001/jamanetworkopen.2022.31878

eMethods.

eFigure 1. Study Population Flow Chart

eFigure 2. Associations of Cardiovascular and Noncardiovascular Dysfunctions with Incident HF

eTable 1. Systems Evaluated in Risk Models for Heart Failure

eTable 2. Cardiovascular and Noncardiovascular Characteristics by Incident HF Subtypes in Women

eTable 3. Cardiovascular and Noncardiovascular Characteristics by Incident HF Subtypes in Men

eTable 4. Associations of Cardiovascular and Noncardiovascular Measures With Incident HF Events in Unipredictor Multivariable Models Additionally Adjusting For Income Status and Education Attainment

eTable 5. Associations of Cardiovascular and Noncardiovascular Measures With Incident HF Events in Multipredictor Models

eTable 6. Cox Regression Models for HF, HFpEF, and HFrEF with Multiple Imputation

eTable 7. Associations of Cardiovascular and Noncardiovascular Measures With Incident HF Events in Multipredictor Models Excluding Participants With a Baseline LVEF Less Than 50%

eTable 8. Exploration of Potential Interactions of Organ Function Measures with Sex for Incident HF

eTable 9. Stratified Cox Regression Models for HF, HFpEF, and HFrEF by Sex Categories

eReferences.

This supplemental material has been provided by the authors to give readers additional information about their work.

© 2022 Zhao L et al. *JAMA Network Open.*

eMethods

1. **Derivation of echocardiographic measures**

2. **Measurements of non-cardiac plasma markers**

3. **Definition of social determinants of health**

4. **Details of multiple imputation by chained equations**

1. Derivation of echocardiographic measures

Mean wall thickness (MWT) was calculated as the mean of the septal and posterior wall thicknesses. Relative wall thickness (RWT) was calculated using the following formula: RWT=(2*PWT)/ LVEDD where PWT is posterior wall thickness and LVEDD is left ventricular end-diastolic dimension. Fractional shortening was defined as follows: [(LVEDD–LVESD)/ (LVEDD)] *100% where LVESD is LV end-systolic dimension. LV mass (LVM) was calculated using the formula: $0.89(1.04* ((SWT+ LVEDD+ PWT)^3 – (LVEDD)^3) + 0.6$ where SWT is septal wall thickness, and indexed to BSA to derive LV mass index (LVMI) [1]. LVEF was calculated using Teichholz method: $((7/(2.4+LVEDD) *(LVEDD)^3)- (7/(2.4+ LVESD)* (LVESD)^3))/ (7/(2.4+ LVEDD)* (LVEDD)^3)*100\%$. When a quantitative LVEF was not available, semiquantitative measures were used (LVEF estimated to nearest 5%). Valvular disease was graded qualitatively as previously described [2].

© 2022 Zhao L et al. *JAMA Network Open.*

2. Measurements of non-cardiac plasma markers

Plasma adiponectin was measured in nanograms per milliliter, by an ELISA system (R&D Systems; Minneapolis, MN, USA) [3]. Leptin was analyzed with Human Leptin RIA kit (LINCO Research, St Charles, MI, USA) [3]. HbA1c was measured using a TOSOH high-performance liquid chromatography system at the University of Minnesota Department of Laboratory Medicine and Pathology [2]. High sensitivity C-reactive protein (hs-CRP) was measured using an immunoturbidimetric CRP-Latex assay from Kamiya Biomedical Company following manufacturer's high-sensitivity protocol [4].

© 2022 Zhao L et al. *JAMA Network Open.*

3. Definition of social determinants of health

Income status was categorized into 4 levels: poor defined as income less than poverty level; lower-middle defined as income 1 to 1.5 times the poverty level; upper-middle defined as income greater than 1.5 but less than 3.5 times the poverty level; and affluent defined as income no less than 3.5 times the poverty level.[5] Educational attainment was based on self-report of years of schooling completed: less than high school; high school graduate or general equivalency; and vocational school, trade school or college graduate.[5] The neighborhood problems scale comprised 6 items scored from 1 (not really a problem) to 4 (very serious problem): excessive noise; heavy traffic or speeding cars; lack of access to adequate food and/or shopping; lack of parks and playground; trash and litter; and no sidewalks and poorly maintained sidewalks.[6]

© 2022 Zhao L et al. *JAMA Network Open.*

4. Details of multiple imputation by chained equations

Imputation was performed for 11 clinical and echocardiographic measures (LVMI, LVEF, LAD, PP, ppFEV1, eGFR, HbA1c, Waist, hypertension, diabetes, smoking status) with missing data and was based on linear regression using 9 baseline clinical variables (age, gender, BMI, SBP, adiponectin, leptin, hsCRP, HTN meds, DM meds) and 3 echocardiographic measures (LVEDD, MWT, RWT) as predictor variables and was derived over 40 imputations.

© 2022 Zhao L et al. *JAMA Network Open.*

eFigure 1. Study Population Flow Chart

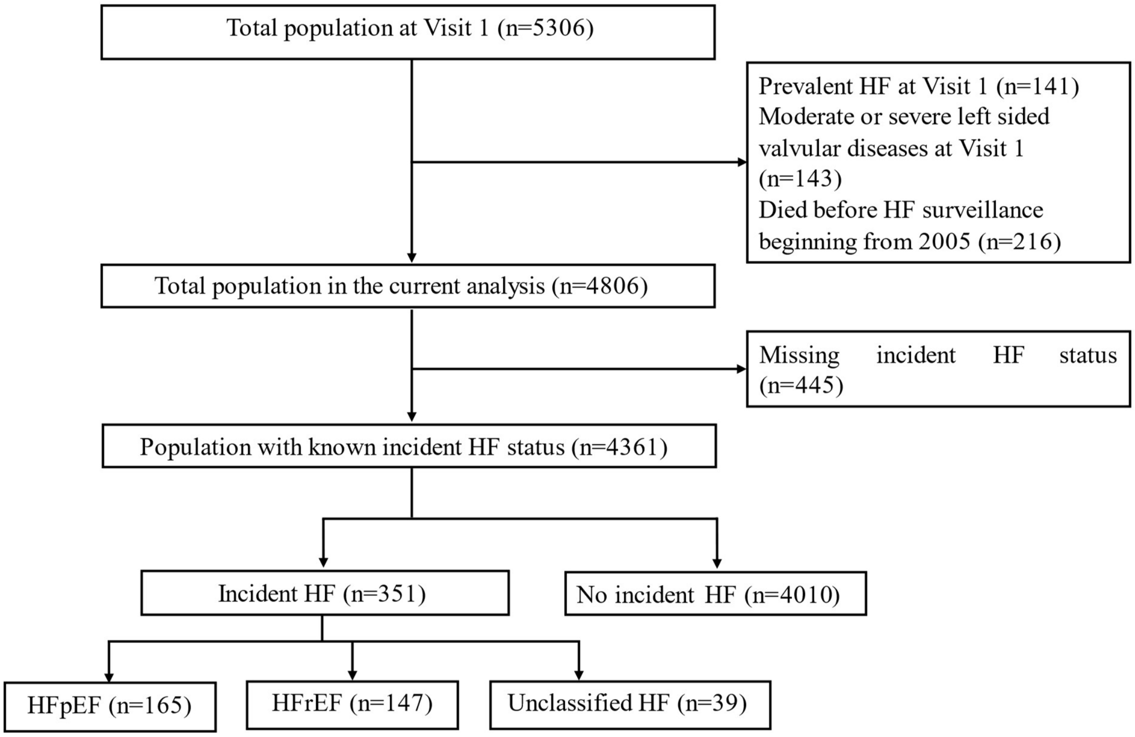

The flow chart of the eligibility of the study population. HF, heart failure.

© 2022 Zhao L et al. *JAMA Network Open.*

eFigure 2. Associations of Cardiovascular and Noncardiovascular Dysfunctions With Incident HF

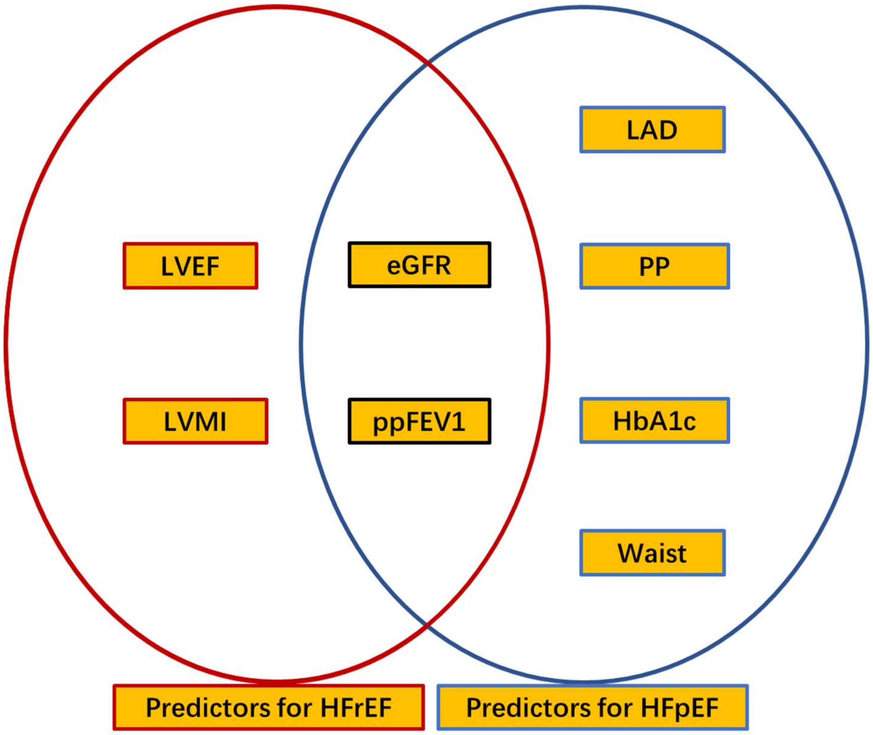

Subclinical impairments in both cardiovascular and non-cardiovascular organ function differentially associated with risk of incident HFpEF and HFrEF in Black Adults.

HF = heart failure; HFrEF = HF with reduced ejection fraction; HFpEF = HF with preserved ejection fraction; LVEF = left ventricular ejection fraction; LVMI = left ventricular mass index; ppFEV1 = percent predicted forced expiration volume in 1 second; HbA1c = hemoglobin A1c; LAD = left atrial diameter; PP = pulse pressure; waist = waist circumference; eGFR = estimated glomerular filtration rate.

© 2022 Zhao L et al. *JAMA Network Open.*

eTable 1. Systems Evaluated in Risk Models for Heart Failure

Systems	Variables
LV structure	LV mass index (LVMI), diastolic LV diameter (LVEDD), LV mean wall thickness (MWT), LV relative wall thickness (RWT)
LV systolic function	LV ejection fraction (LVEF), fractional shortening (not used due to high missingness)
LV diastolic function	E/A ratio, LA diameter (LAD)
Systemic arterial function	Pulse pressure (PP)
Renal function	eGFR, random spot urine albumin (not used due to high missingness)
Pulmonary function	Percent predicted forced expiration volume in 1 second (ppFEV1), Percent predicted forced vital capacity (ppFVC), FEV1/FVC
Dysglycemia	Diabetes, diabetes medicine (DM Meds), HbA1c
Body composition	body mass index (BMI), waist circumference (waist), adiponectin, leptin
Inflammation	hs-CRP

© 2022 Zhao L et al. *JAMA Network Open.*

eTable 2. Cardiovascular and Noncardiovascular Characteristics by Incident HF Subtypes in Women

	No HF	HFpEF	HFrEF	Unclassified HF	P value
	n=2555	n=118	n=75	n=28	
Age (years)	54±12	66±10	64±10	67±10	p<0.001
**LV Structure**					
LVEDD (mm)	47.3±3.9	47.7±4.4	49.2±4.3	48.1±5.5	p<0.001
MWT (mm)	8.5±1.3	9.2±1.4	8.9±1.5	9.3±1.0	p<0.001
RWT	0.35±0.06	0.38±0.07	0.36±0.06	0.37±0.05	p<0.001
LVMI (g/m^2)	70.3±17.1	77.8±19.7	80.2±20.8	81.5±21.9	p<0.001
**LV Systolic Function**					
LVEF (%)	64.4±7.4	64.7±7.3	59.9±9.1	63.5±8.6	p<0.001
**LV Diastolic Function**					
LA diameter (mm)	34.2±4.0	36.0±4.4	35.5±6.1	35.7±3.5	p<0.001
E/A	1.09±0.34	0.92±0.28	0.96±0.27	0.97±0.20	p<0.001
**Systemic Arterial Function Related**					
Hypertension	1407(55.1%)	100(84.7%)	56(74.7%)	22(78.6%)	p<0.001
HTN Meds	1332(52.5%)	99(83.9%)	55(73.3%)	22(78.6%)	p<0.001
SBP (mmHg)	126±16	134±20	133±16	140±21	p<0.001
Pulse Pressure (mmHg)	51±14	62±18	60±16	67±22	p<0.001
**Pulmonary Function**					
ppFEV1 (%)	94±17	88±20	91±19	95±19	p<0.001
ppFVC (%)	93±18	88±20	94±28	93±22	p=0.039
FEV1/FVC	0.82±0.08	0.79±0.09	0.78±0.11	0.80±0.06	p<0.001
**Renal Function**					
eGFR (ml/(min.1.73m^2))	97±20	80±26	81±30	86±24	p<0.001
**Dysglycemia Related**					
Diabetes	528(20.9%)	62(53.0%)	40(53.3%)	11(39.3%)	p<0.001
HbA1c (%)	5.9±1.2	6.8±2.0	6.6±1.8	6.3±1.2	p<0.001
DM Meds	345(13.6%)	49(41.9%)	31(41.3%)	10(35.7%)	p<0.001
**Obesity Related**					
BMI (kg/m^2)	32.7±7.4	34.9±7.7	31.8±6.9	33.5±7.1	p=0.01
Waist (cm)	100±16	109±16	102±17	105±17	p<0.001
Adiponectin (ng/ml)	4890 [3189,7364]	5627 [3611,9424]	5416 [3416,7887]	5319 [4166,11735]	p=0.01
Leptin (ng/ml)	32.9[22.3,46.5]	39.2[24.9,60.8]	31.5[20.1,44.1]	35.0[20.6,57.8]	p=0.009
**Inflammation**					
hsCRP (mg/L)	0.34[0.14,0.68]	0.52[0.20,0.88]	0.35[0.19,0.81]	0.25[0.13,0.54]	p=0.006
**Smoking Status**					
Ever Smoker	604(23.7%)	43(36.4%)	26(34.7%)	8(28.6%)	p=0.002

© 2022 Zhao L et al. *JAMA Network Open.*

**Disease History**					
CHD (MI) history	90(3.5 %)	9(7.6 %)	13(17.3%)	3(10.7%)	p<0.001
**Social Determinations of Health**					
**Income Status**					p<0.001
Affluent	345(15.9%)	26(27.1%)	13(20.3%)	6(25.0%)	
Upper-middle	550(25.4%)	40(41.7%)	22(34.4%)	6(25.0%)	
Lower-middle	661(30.5%)	20(20.8%)	19(29.7%)	4(16.7%)	
Poor	613(28.3%)	10(10.4%)	10(15.6%)	8(33.3%)	
**Education Attainment Categorization**					p<0.001
Attended vocational school, trade school, or college	374(14.7%)	55(46.6%)	26(34.7%)	13(46.4%)	
High school graduate/GED	514(20.2%)	24(20.3%)	17(22.7%)	4(14.3%)	
Less than high school	1660(65.1%)	39(33.1%)	32(42.7%)	11(39.3%)	
**Neighborhood Problems**	1.56±0.18	1.66±0.16	1.60±0.15	1.60±0.19	p<0.001

HF, heart failure; HFpEF, HF with preserved ejection fraction; HFpEF, HF with reduced ejection fraction; LVEDD, left ventricular end-diastolic dimension; MWT, left ventricular mean wall thickness; RWT, left ventricular relative wall thickness; LVMI, left ventricular mass index; LVEF, left ventricular ejection fraction; LA, left atrium; HTN, hypertension; SBP, systolic blood pressure; ppFEV1, percent predicted forced expiration volume in 1 second; ppFVC, percent predicted forced vital capacity; eGFR, estimated global filtration rate; HbA1c, hemoglobin A1c; Waist, waist circumference; hsCRP, high sensitivity C reactive protein; CHD, coronary heart disease; MI, myocardial infarction; GED, general educational development.

© 2022 Zhao L et al. *JAMA Network Open.*

eTable 3. Cardiovascular and Noncardiovascular Characteristics by Incident HF Subtypes in Men

	No HF	HFpEF	HFrEF	Unclassified HF	P value
	n=1458	n=45	n=71	n=11	
Age (years)	53±12	61±11	61±14	73±4	p<0.001
**LV Structure**					
LVEDD (mm)	49.6±4.0	48.4±5.0	51.9±5.9	50.8±3.9	p<0.001
MWT (mm)	9.1±1.3	10.1±1.8	9.5±1.6	9.8±1.5	p<0.001
RWT	0.36±0.06	0.42±0.11	0.36±0.07	0.36±0.05	p<0.001
LVMI (g/m^2)	75.6±17.5	81.3±19.5	86.2±24.2	87.4±24.0	p<0.001
**LV Systolic Function**					
LVEF (%)	61.5±8.0	63.4±6.8	55.9±11.0	61.3±11.3	p<0.001
**LV Diastolic Function**					
LA diameter (mm)	35.9±4.2	38.5±4.5	37.0±5.5	38.3±4.8	p<0.001
E/A	1.13±0.35	0.98±0.32	0.99±0.34	0.88±0.21	p<0.001
**Systemic Arterial Function Related**					
Hypertension	686(47.1%)	36(80.0%)	52(73.2%)	9(81.8%)	p<0.001
HTN Meds	578(40.0%)	32(71.1%)	45(64.3%)	8(80.0%)	p<0.001
SBP (mmHg)	127±16	138±19	133±18	132±18	p<0.001
Pulse Pressure (mmHg)	49±13	60±17	55±14	58±13	p<0.001
**Pulmonary Function**					
ppFEV1 (%)	91±16	83±17	84±18	74±22	p<0.001
ppFVC (%)	90±15	82±16	85±14	74±19	p<0.001
FEV1/FVC	0.80±0.09	0.78±0.08	0.77±0.10	0.74±0.07	p=0.007
**Renal Function**					
eGFR (ml/(min.1.73m^2))	95±19	86±22	90±23	78±17	p<0.001
**Dysglycemia Related**					
Diabetes	259(17.9%)	24(54.5%)	28(40.0%)	6(54.5%)	p<0.001
HbA1c (%)	5.9±1.2	6.9±2.0	6.6±2.1	7.1±2.2	p<0.001
DM Meds	147(10.2%)	20(44.4%)	21(30.0%)	4(40.0%)	p<0.001
**Obesity Related**					
BMI (kg/m^2)	29.6±5.8	32.3±6.9	30.9±5.5	30.2±5.1	p=0.006
Waist (cm)	100±15	110±16	106±14	103±9	p<0.001
Adiponectin (ng/ml)	3040 [2010,4806]	3648 [1816,5937]	3683 [2571,5812]	5922 [3306,9642]	p=0.002
Leptin (ng/ml)	8.2[4.7,13.7]	13.6[7.5,21.1]	9.2[4.4,15.9]	11.4[5.2,18.5]	p=0.007
**Inflammation**					
hsCRP (mg/L)	0.16[0.07,0.35]	0.25[0.14,0.42]	0.19[0.10,0.42]	0.29[0.09,0.43]	p=0.05
**Smoking Status**					
Ever Smoker	607(41.7%)	25(55.6%)	35(50.0%)	5(45.5%)	p=0.16

© 2022 Zhao L et al. *JAMA Network Open.*

**Disease History**					
CHD (MI) history	79(5.4 %)	10(22.2%)	7(9.9 %)	2(18.2%)	p<0.001
**Social Determinations of Health**					
**Income Status**					p=0.002
Affluent	106(8.6 %)	4(10.3%)	9(15.3%)	1(12.5%)	
Upper-middle	233(18.8%)	11(28.2%)	22(37.3%)	2(25.0%)	
Lower-middle	379(30.6%)	10(25.6%)	14(23.7%)	5(62.5%)	
Poor	520(42.0%)	14(35.9%)	14(23.7%)	0(0.0 %)	
**Education Attainment Categorization**					p<0.001
Attended vocational school, trade school, or college	229(15.8%)	11(24.4%)	25(35.7%)	7(63.6%)	
High school graduate/GED	275(18.9%)	9(20.0%)	13(18.6%)	3(27.3%)	
Less than high school	949(65.3%)	25(55.6%)	32(45.7%)	1(9.1 %)	
**Neighborhood Problems**	1.54±0.19	1.62±0.18	1.58±0.18	1.63±0.16	p=0.009

HF, heart failure; HFpEF, HF with preserved ejection fraction; HFpEF, HF with reduced ejection fraction; LVEDD, left ventricular end-diastolic dimension; MWT, left ventricular mean wall thickness; RWT, left ventricular relative wall thickness; LVMI, left ventricular mass index; LVEF, left ventricular ejection fraction; LA, left atrium; HTN, hypertension; SBP, systolic blood pressure; ppFEV1, percent predicted forced expiration volume in 1 second; ppFVC, percent predicted forced vital capacity; eGFR, estimated global filtration rate; HbA1c, hemoglobin A1c; Waist, waist circumference; hsCRP, high sensitivity C reactive protein; CHD, coronary heart disease; MI, myocardial infarction; GED, general educational development.

© 2022 Zhao L et al. *JAMA Network Open.*

eTable 4. Associations of Cardiovascular and Noncardiovascular Measures With Incident HF Events in Unipredictor Multivariable Models Additionally Adjusting for Income Status and Education Attainment

	Hazard Ratio for Incident HF		
	HR	(95% CI)	P value
**LV Structure**			
LVEDD (mm)	1.34	(1.19, 1.50)	<0.001
MWT (mm)	1.17	(1.05, 1.31)	0.005
RWT	0.99	(0.88, 1.11)	0.91
LVMI (g/m^2)	1.23	(1.11, 1.35)	<0.001
**LV Systolic Function**			
LVEF (%)	1.30	(1.17, 1.44)	<0.001
**LV Diastolic Function**			
LA diameter (mm)	1.23	(1.10, 1.37)	<0.001
E/A ratio	0.99	(0.85, 1.17)	0.95
**Systemic Arterial Function**			
SBP (mmHg)	1.12	(1.00, 1.26)	0.054
Pulse Pressure (mmHg)	1.16	(1.03, 1.30)	<0.013
**Pulmonary Function**			
ppFEV1 (%)	1.29	(1.15, 1.45)	<0.001
ppFVC (%)	1.16	(1.03, 1.31)	0.014
FEV1/FVC	1.16	(1.04, 1.29)	0.006
**Renal Function**			
eGFR (ml/(min.1.73m^2))	1.29	(1.12, 1.48)	<0.001
**Dysglycemia Related**			
HbA1c (%)	1.21	(1.10, 1.35)	<0.001
**Obesity Related**			
BMI (kg/m^2)	1.23	(1.09, 1.39)	0.001
Waist (cm)	1.35	(1.19, 1.52)	<0.001
Adiponectin (ng/ml)	1.22	(1.08, 1.38)	0.002
Leptin (ng/ml)	1.23	(1.03, 1.47)	0.021
**Inflammation**			
hsCRP (mg/L)	1.13	(0.99, 1.28)	0.07

The models were adjusted for age, sex, education attainment, income level, hypertension, diabetes, smoking status, and coronary heart disease (myocardial infarction) history.

© 2022 Zhao L et al. *JAMA Network Open.*

eTable 5. Associations of Cardiovascular and Noncardiovascular Measures With Incident HF Events in Multipredictor Models

	HF (n=3806, events=287)			HFpEF (n=3806, events=131)			HFrEF (n=3806, events=123)			HFrEF vs HFpEF
	HR	(95% CI)	P value	HR	(95% CI)	P value	HR	(95% CI)	P value	P value
LVMI (g/m^2)	1.17	(1.05, 1.29)	0.004	1.03	(0.87, 1.21)	0.74	1.25	(1.07, 1.46)	0.005	0.08
LVEF (%)	1.27	(1.15, 1.42)	<0.001	1.01	(0.85, 1.19)	0.94	1.65	(1.42, 1.91)	<0.001	<0.001
LA diameter (mm)	1.16	(1.03, 1.31)	0.012	1.23	(1.03, 1.47)	0.021	1.08	(0.90, 1.29)	0.43	0.34
PP (mmHg)	1.15	(1.03, 1.29)	0.012	1.23	(1.05, 1.44)	0.009	1.05	(0.87, 1.27)	0.61	0.24
ppFEV1 (%)	1.20	(1.08, 1.35)	0.001	1.22	(1.04, 1.43)	0.017	1.19	(1.00, 1.42)	0.047	0.86
eGFR (ml/(min.1.73m^2))	1.30	(1.14, 1.47)	<0.001	1.43	(1.19, 1.72)	<0.001	1.27	(1.04, 1.55)	0.022	0.5
HbA1c (%)	1.21	(1.08, 1.36)	0.001	1.25	(1.07, 1.45)	0.005	1.18	(0.99, 1.41)	0.06	0.73
Waist (cm)	1.22	(1.08, 1.39)	0.002	1.41	(1.18, 1.69)	<0.001	1.05	(0.85, 1.30)	0.66	0.031

The models were adjusted for age, sex, hypertension, diabetes, smoking status, and coronary heart disease (myocardial infarction) history. All continuous variables were standardized. The HRs for LVEF, ppFEV1and eGFR were inverted to facilitate the comparison of the magnitude of associations of the cardiovascular and non-cardiovascular measures with the outcome.

HF, heart failure; HFpEF, HF with preserved ejection fraction; HFpEF, HF with reduced ejection fraction; HR, hazard ratio; CI, confidence interval; LVMI, left ventricular mass index; LVEF, left ventricular ejection fraction; LA, left atrium; PP, pulse pressure; ppFEV1, percent predicted forced expiration volume in 1 second; eGFR, estimated global filtration rate; HbA1c, hemoglobin A1c; Waist, waist circumference.

© 2022 Zhao L et al. *JAMA Network Open.*

eTable 6. Cox Regression Models for HF, HFpEF, and HFrEF With Multiple Imputation

	HF(n=4361， events=348)			HFpEF(n=4361， events=163)			HFrEF(n=4361， events=146)		
	HR	(95% CI)	P value	HR	(95% CI)	P value	HR	(95% CI)	P value
LVMI	1.15	(1.04, 1.26)	0.005	1.03	(0.89, 1.19)	0.71	1.23	(1.07, 1.42)	0.005
LVEF	1.27	(1.15, 1.40)	<0.001	1.00	(0.85, 1.17)	0.98	1.62	(1.41, 1.87)	<0.001
LAD	1.15	(1.03, 1.29)	0.012	1.23	(1.04, 1.46)	0.014	1.05	(0.88, 1.25)	0.58
PP	1.16	(1.05, 1.29)	0.005	1.23	(1.06, 1.42)	0.007	1.06	(0.89, 1.27)	0.51
ppFEV1	1.22	(1.09, 1.37)	<0.001	1.25	(1.06, 1.47)	0.006	1.19	(1.00, 1.42)	0.05
eGFR	1.28	(1.14, 1.45)	<0.001	1.43	(1.20, 1.69)	<0.001	1.24	(1.01, 1.50)	0.035
HbA1c	1.22	(1.09, 1.35)	<0.001	1.26	(1.08, 1.46)	0.002	1.19	(1.00, 1.41)	0.046
Waist	1.21	(1.07, 1.38)	0.002	1.39	(1.17, 1.64)	<0.001	1.05	(0.85, 1.29)	0.65

Imputation was performed for 11 clinical and echocardiographic measures (LVMI, LVEF, LAD, PP, ppFEV1, eGFR, HbA1c, Waist, hypertension, diabetes, smoking status) with missing data and was based on linear regression using 9 baseline clinical variables (age, gender, BMI, SBP, adiponectin, leptin, hsCRP, HTN meds, DM meds) and 3 echocardiographic measures (LVEDD, MWT, RWT) as predictor variables and was derived over 40 imputations. Models were adjusted for age, gender, hypertension, diabetes, smoking status and CHD history.

HF, heart failure; HFpEF, HF with preserved ejection fraction; HFpEF, HF with reduced ejection fraction; LVEDD, left ventricular end-diastolic dimension; MWT, left ventricular mean wall thickness; RWT, left ventricular relative wall thickness; LVMI, left ventricular mass index; LVEF, left ventricular ejection fraction; LA, left atrium; HTN, hypertension; SBP, systolic blood pressure; ppFEV1, percent predicted forced expiration volume in 1 second; ppFVC, percent predicted forced vital capacity; eGFR, estimated global filtration rate; HbA1c, hemoglobin A1c; Waist, waist circumference; hsCRP, high sensitivity C reactive protein; HR, hazard ratio; CI, confidence interval.

© 2022 Zhao L et al. *JAMA Network Open.*

eTable 7. Associations of Cardiovascular and Noncardiovascular Measures With Incident HF Events in Multipredictor Models Excluding Participants With a Baseline LVEF of Less Than 50%

	HFpEF (n=3622, events=130)			HFrEF (n=3622, events=102)		
	HR	(95% CI)	P value	HR	(95% CI)	P value
LVMI (g/m^2)	1.05	(0.90, 1.24)	0.53	1.15	(0.96, 1.37)	0.13
LVEF (%)	1.12	(0.95, 1.32)	0.17	1.57	(1.29, 1.91)	<0.001
LA diameter (mm)	1.25	(1.04, 1.49)	0.016	1.18	(0.96, 1.44)	0.13
PP (mmHg)	1.24	(1.06, 1.43)	0.007	1.11	(0.91, 1.36)	0.31
ppFEV1 (%)	1.21	(1.03, 1.43)	0.020	1.20	(0.99, 1.45)	0.06
eGFR (ml/min1.73m^2)	1.42	(1.18, 1.71)	<0.001	1.27	(1.02, 1.58)	0.034
HbA1c (%)	1.23	(1.05, 1.44)	0.010	1.20	(1.00, 1.45)	0.053
Waist (cm)	1.42	(1.19, 1.70)	<0.001	1.02	(0.81, 1.29)	0.85

The models were adjusted for age, sex, hypertension, diabetes, smoking status, and coronary heart disease (myocardial infarction) history. All continuous variables were standardized. The HRs for LVEF, ppFEV1and eGFR were inverted to facilitate the comparison of the magnitude of associations of the cardiovascular and non-cardiovascular measures with the outcome.

HF, heart failure; HFpEF, HF with preserved ejection fraction; HFpEF, HF with reduced ejection fraction; HR, hazard ratio; CI, confidence interval; LVMI, left ventricular mass index; LVEF, left ventricular ejection fraction; LA, left atrium; PP, pulse pressure; ppFEV1, percent predicted forced expiration volume in 1 second; eGFR, estimated global filtration rate; HbA1c, hemoglobin A1c; Waist, waist circumference.

© 2022 Zhao L et al. *JAMA Network Open.*

eTable 8. Exploration of Potential Interactions of Organ Function Measures With Sex for Incident HF

	HF (n=3808; events=261)			HFpEF (n=3808; events=121)			HFrEF (n=3808; events=115)		
	HR	95% CI	P	HR	95% CI	P	HR	95% CI	P
LVMI	1.18	(1.04, 1.35)	0.011	1.10	(0.91, 1.33)	0.33	1.23	(0.99, 1.52)	0.06
LVEF	1.30	(1.13, 1.50)	<0.001	1.13	(0.92, 3.33)	0.24	1.65	(1.33, 2.05)	<0.001
LAD	1.18	(1.01, 1.38)	0.036	1.21	(0.97, 1.33)	0.09	1.17	(0.90, 1.53)	0.24
PP	1.18	(1.04, 1.34)	0.009	1.20	(1.01, 1.33)	0.038	1.09	(0.87, 1.36)	0.48
ppFEV1	1.22	(1.06, 1.40)	0.004	1.29	(1.07, 2.33)	0.008	1.21	(0.96, 1.53)	0.11
eGFR	1.43	(1.24, 1.66)	<0.001	1.55	(1.26, 4.33)	<0.001	1.50	(1.18, 1.91)	0.001
HbA1c	1.36	(1.23, 1.52)	<0.001	1.40	(1.22, 3.33)	<0.001	1.39	(1.16, 1.67)	<0.001
Waist	1.29	(1.11, 1.50)	0.001	1.52	(1.24, 2.33)	<0.001	1.00	(0.76, 1.32)	0.99
LVMI*Male	0.98	(0.79, 1.22)	0.88	0.81	(0.56, 9.33)	0.27	1.06	(0.78, 1.45)	0.71
LVEF*Male	0.98	(0.79, 1.21)	0.85	0.71	(0.49, 9.33)	0.06	1.01	(0.75, 1.37)	0.93
LAD*Male	1.09	(0.85, 1.38)	0.51	1.35	(0.91, 0.33)	0.14	0.91	(0.64, 1.30)	0.62
PP*Male	0.96	(0.77, 1.21)	0.73	1.24	(0.89, 9.33)	0.20	0.95	(0.67, 1.35)	0.78
ppFEV1*Male	1.08	(0.86, 1.36)	0.52	0.94	(0.66, 0.33)	0.75	1.07	(0.75, 1.51)	0.72
eGFR*Male	0.75	(0.58, 0.98)	0.032	0.78	(0.52, 7.33)	0.23	0.66	(0.45, 0.96)	0.031
HbA1c*Male	0.97	(0.82, 1.16)	0.75	0.98	(0.75, 9.33)	0.91	0.94	(0.72, 1.21)	0.62
Waist *Male	0.90	(0.68, 1.18)	0.43	0.79	(0.52, 9.33)	0.26	1.19	(0.80, 1.77)	0.39
Age	1.07	(1.06, 1.09)	<0.001	1.07	(1.05, 0.33)	<0.001	1.05	(1.03, 1.07)	<0.001
Male	1.11	(0.79, 1.56)	0.54	0.79	(0.44, 1.33)	0.43	1.49	(0.91, 2.45)	0.11

HF, heart failure; HFpEF, heart failure with preserved ejection fraction; HFrEF, heart failure with reduced ejection fraction; HR, hazard ratio; CI, confidence interval; LVEF, left ventricular ejection fraction; LVMI, left ventricular mass index; ppFEV1, percent predicted forced expiration volume in 1 second; HbA1c, hemoglobin A1c; LAD, left atrial diameter; PP, pulse pressure; waist, waist circumference; eGFR, estimated glomerular filtration rate.

© 2022 Zhao L et al. *JAMA Network Open.*

eTable 9. Stratified Cox Regression Models for HF, HFpEF, and HFrEF by Sex Categories

	HF-Female (n=2404; events=177)				HF-Male (n=1408; events=110)			
**HF overall**	HR	95% CI		P	HR	95% CI		P
LVMI	1.18	1.04	1.34	0.013	1.17	0.98	1.39	0.08
LVEF	1.30	1.13	1.50	<0.001	1.27	1.08	1.49	0.003
LAD	1.18	1.01	1.38	0.036	1.28	1.06	1.55	0.009
PP	1.17	1.03	1.33	0.016	1.15	0.94	1.41	0.17
ppFEV1	1.22	1.07	1.40	0.004	1.33	1.11	1.60	0.003
eGFR	1.41	1.21	1.64	<0.001	1.10	0.87	1.40	0.43
HbA1c	1.36	1.23	1.52	<0.001	1.33	1.16	1.52	<0.001
Waist	1.29	1.11	1.51	0.001	1.14	0.91	1.43	0.24
	HFpEF-Female (n=2404; events=92)				HFpEF-Male (n=1408; events=39)			
**HFpEF**	HR	95% CI		P	HR	95% CI		P
LVMI	1.09	0.90	1.32	0.36	0.89	0.65	1.23	0.49
LVEF	1.15	0.93	1.40	0.19	0.80	0.59	1.07	0.17
LAD	1.21	0.97	1.50	0.08	1.61	1.15	2.26	0.006
PP	1.16	0.98	1.39	0.09	1.63	1.19	2.25	0.003
ppFEV1	1.30	1.08	1.57	0.01	1.27	0.92	1.74	0.14
eGFR	1.47	1.20	1.82	0.00	1.35	0.93	1.97	0.12
HbA1c	1.41	1.23	1.62	0.00	1.37	1.10	1.71	0.005
Waist	1.54	1.25	1.88	0.00	1.16	0.81	1.66	0.42
	HFrEF-Female (n=2404; events=62)				HFrEF-Male (n=1408; events=61)			
**HFrEF**	HR	95% CI		P	HR	95% CI		P
LVMI	1.23	0.99	1.52	0.06	1.30	1.03	1.63	0.025
LVEF	1.64	1.32	2.03	<0.001	1.68	1.37	2.07	<0.001
LAD	1.16	0.89	1.52	0.27	1.09	0.86	1.38	0.49
PP	1.11	0.88	1.40	0.37	1.00	0.75	1.34	0.99
ppFEV1	1.20	0.94	1.52	0.14	1.29	1.00	1.66	0.048
eGFR	1.53	1.20	1.96	0.001	0.95	0.68	1.32	0.76
HbA1c	1.39	1.15	1.66	<0.001	1.30	1.08	1.56	0.005
Waist	0.99	0.75	1.31	0.96	1.19	0.89	1.59	0.23

Models were adjusted for age. HF, heart failure; HFpEF, heart failure with preserved ejection fraction; HFrEF, heart failure with reduced ejection fraction; HR, hazard ratio; CI, confidence interval; LVEF, left ventricular ejection fraction; LVMI, left ventricular mass index; ppFEV1, percent predicted FEV1; HbA1c, hemoglobin A1c; LAD, left atrial diameter; PP, pulse pressure; waist, waist circumference; eGFR, estimated glomerular filtration rate.

© 2022 Zhao L et al. *JAMA Network Open.*

eReferences.

1. Du Bois D, Du Bois EF. A formula to estimate the approximate surface area if height and weight be known. 1916. *Nutrition (Burbank, Los Angeles County, Calif).* 1989;5(5):303-311; discussion 312-303.
2. Carpenter MA, Crow R, Steffes M, et al. Laboratory, reading center, and coordinating center data management methods in the Jackson Heart Study. *Am J Med Sci.* 2004;328(3):131-144.
3. Bidulescu A, Liu J, Chen Z, et al. Associations of adiponectin and leptin with incident coronary heart disease and ischemic stroke in african americans: the jackson heart study. *Front Public Health.* 2013;1:16.
4. Fox ER, Benjamin EJ, Sarpong DF, et al. Epidemiology, heritability, and genetic linkage of C-reactive protein in African Americans (from the Jackson Heart Study). *Am J Cardiol.* 2008;102(7):835-841.
5. Forde AT, Sims M, Muntner P, et al. Discrimination and Hypertension Risk Among African Americans in the Jackson Heart Study. *Hypertension.* 2020;76(3):715-723.
6. Gebreab SY, Hickson DA, Sims M, et al. Neighborhood social and physical environments and type 2 diabetes mellitus in African Americans: The Jackson Heart Study. *Health Place.* 2017;43:128-137.

© 2022 Zhao L et al. *JAMA Network Open.*
